# A case report of a drug‐induced liver injury (DILI) caused by multiple antidepressants with causality established by the updated Roussel Uclaf causality assessment method (RUCAM) and in vitro testing

**DOI:** 10.1002/ccr3.3348

**Published:** 2020-09-21

**Authors:** Miguel González‐Muñoz, Jaime Monserrat Villatoro, Eva Marín‐Serrano, Stefan Stewart, Belén Bardón Rivera, Jesús Marín, Lucía Martínez de Soto, Enrique Seco Meseguer, Elena Ramírez

**Affiliations:** ^1^ Immunology Department La Paz‐Cantoblanco‐Carlos III University Hospital IdiPaz Madrid España; ^2^ Clinical Pharmacology Department Facultad de Medicina La Paz‐Cantoblanco‐Carlos III University Hospital IdiPaz Universidad Autónoma de Madrid Madrid España; ^3^ Gastroenterology and Hepatology Department La Paz‐Cantoblanco‐Carlos III University Hospital IdiPaz Madrid España; ^4^ Psiquiatry Department La Paz‐Cantoblanco‐Carlos III University Hospital, IdiPaz Madrid España

**Keywords:** antidepressants, causality assessment, drug‐induced liver injury (DILI), lymphocyte transformation test (LTT), pharmacovigilance, Roussel Uclaf Causality Assessment Method (RUCAM)

## Abstract

A 56‐year‐old female patient was hospitalized because of a lack of response and poor tolerance to multiple antidepressants, which included an episode of DILI. During hospitalization, the patient suffered another episode of DILI. Causality was assessed both by RUCAM and Lymphocyte Transformation Test, allowing to identify a safer medication.

## Graphical abstract

Roussel Uclaf Causality Assessment Method (RUCAM) is the great value to diagnosis DILI. In polypharmacy settings, laboratory‐based confirmatory assays may be an important option to diagnose the culprit drug. In this case, the lymphocyte transformation test was applied for drugs with RUCAM score ≥ 6 to explore causality and the cross reactivity.

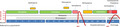

## 1 INTRODUCTION

Drug‐Induced Liver Injury (DILI) is the fourth leading cause of liver damage in Western countries, increasingly becoming a matter of concern for drug prescription.^1^ Despite data on antidepressant‐induced liver injury being scarce, 0.5%‐3% of patients treated with antidepressants may develop hepatitis,^2^, ^3^ being the most susceptible population the elderly and those with polypharmacy.^2^ Liver damage is in most cases idiosyncratic and unpredictable, and it is generally unrelated to drug dosage.^2^ Patients with DILI by antidepressants should be presumed to have increased risk of developing DILI with the same antidepressant or with any other antidepressant that may display cross‐toxicity, limiting therapeutic options.

## 2 CASE DESCRIPTION

**FIGURE 1:**
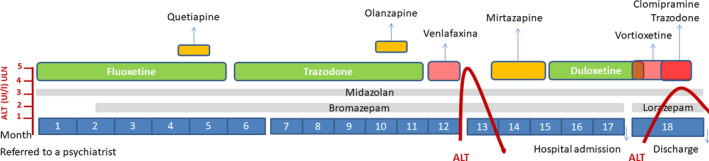
Timeline of prescribed medication and the two drug‐induced liver injury (DILI) episodes. DILI, following case definition,^1^ is represented by times the upper limit of normality (ULN) of alanine aminotransferase (ALT) titers. Alkaline phosphatase, total bilirubin, and prothrombin activity remained consistently under the ULN during the episodes

A 56‐year‐old Caucasian female patient with a medical history of recurring depression disorder, no alcohol consumption, no toxic substances abuse was referred by her psychiatrist for hospital admission because of refractory dysthymia and generalized anxiety disorder as well as a poor tolerance to multiple antidepressant drugs prescribed during a period of 17 months. Before hospitalization, the patient had been prescribed fluoxetine during a 5‐month period followed by trazodone for 6 months, both of them without any effect; quetiapine and olanzapine for a month respectively producing dizziness and fatigue, which led to it being replaced by venlafaxine starting with a daily dose 75 mg for 2 weeks, followed by 150 mg daily for the next 2 weeks, with partial response, eventually being withdrawn after an alteration on liver tests with elevation of alanine aminotransferase (ALT) 175 IU/L (upper limit of normality [ULN], 35) and aspartate aminotransferase (AST) 148 IU/L (ULN, 40), while alkaline phosphatase (AP), total bilirubin (TB), and prothrombin activity (PA) remained consistently under the ULN. The patient recovered a month after venlafaxine was interrupted. After recovery, mirtazapine 7.5 mg daily was used for 2 months producing dizziness, so it was replaced by duloxetine 90 mg daily until hospitalization with good tolerance but no effectiveness. The co‐medications during the period prior to hospital admission were midazolam 7.5 mg at night and bromazepam 1.5 mg in the morning and 3.5 mg in the night. There were no more relevant medical records or previous pharmacological adverse reactions. During hospitalization, the patient was treated with duloxetine in descending dose from 90 to 10 mg daily, from the 1^st^ day to suspension on 9^th^ day; vortioxetine in ascending dose from 5 to 30 mg daily from the 1^st^ day to 9^th^ day and in descending dose until suspension on 21^st^day; clomipramine 75 mg daily and trazodone 100 mg daily, from the 7^th^ to the 21^st^day. Co‐medications during hospitalization were midazolam 7.5 mg daily and lorazepam 1 mg if necessary (3 doses administered), from 1^st^to the 35^th^ day (day of discharge) (Figure 1).

On 9^th^ day of admission, the patient started with nausea, right hypochondrium pain, and a loss of appetite. Liver tests showcased an elevation of ALT up to 73 IU/L (previous, 34 IU/L); AST to 59 IU/L (previous 26 UI/L). The maximum values of ALT and AST were 132 and 73 IU/L (3.7 and 1.8 times the ULN respectively), on 21^st^ day of admission, AP, TB and PA remained under the ULN. Under the clinical suspicion of DILI, the drug causality of DILI episodes was assessed using the updated Roussel Uclaf Causality Assessment Method (RUCAM 2016).^4^, ^5^ In short, venlafaxine (score + 10) at first DILI episode and vortioxetine (score + 10), clomipramine (score + 9), and trazodone (score + 6) at second DILI episode were the drugs involved according to the RUCAM scale (Table 1). Hepatitis A, B, C, E virus, toxoplasma, IgM Cytomegalovirus, and IgM Epstein‐Barr virus antibodies were negative. IgG Cytomegalovirus and IgG Epstein‐Barr virus antibodies were positive. Findings of the abdominal ultrasound performed on day 23 describe a simple hepatic cyst in segment 8 in an otherwise normal liver parenchyma. No enlargement of either the liver or spleen was objectified. No significant renal alterations were found except for a cortical cyst in the right kidney. Gallbladder and bile duct were also preserved. Medications related were suspended, and liver function was totally recovered in a month. Four months after discharge, the patient underwent a fibroscan with no differing results. One month later, a lymphocyte transformation test (LTT) was performed^6^ to evaluate the antidepressant drugs with a related causality (RUCAM score ≥ +6), that is, venlafaxine at first DILI episode and clomipramine, trazodone, and vortioxetine at 2^nd^ DILI episode. Other antidepressants not involved in the adverse reactions were also tested to assess possible future medication (cross reactivity) (Table 1). LTT shows an immune response to venlafaxine and agrees with the causality algorithm. As for the 2^nd^ episode, only clomipramine was able to induce a T‐cell proliferation (Table 1). Three non‐allergic controls did not show proliferative responses (SI < 2) to the antidepressant drugs. Among drugs tested that were not involved in DILI episodes (Table 1), amitriptyline and escitalopram did not trigger T‐cell proliferation in vitro. According to these results, clinicians selected escitalopram as an alternative medication (20 mg a day), and after 3 months, the patient had a positive clinical response with no adverse reactions. In accordance to The Spanish Data Protection law, informed consent signed by the patient was obtained before publishing. A complete adverse reaction report was submitted to the National Health Authorities in Spain (Pharmacovigilance Center in Madrid), number NR‐6246.

**Table 1:** Results of lymphocyte transformation test (LTT) and RUCAM scores

Antidepressant drug	Class	Drug concentration						
		0.1 µg/mL	1 µg/mL	10 µg/mL	50 µg/mL	Result	RUCAM	
		Stimulation index (SD)					Score^a^ 1st	Score^b^ 2nd
Amitriptyline	TCA	0.5 (0.2)	0.5 (0.1)	0.3 (0.1)	0.3 (0.1)	Negative	Not involved	Not involved
Aripiprazole	SNRI	4.2 (0.7)	3.3 (0.4)	2.2 (0.4)	3.8 (0.6)	Positive	Not involved	Not involved
Clomipramine	TCA	0.8 (0.1)	2.3 (0.3)	0.5 (0.1)	0.2 (0.0)	Positive	Not involved	+9
Escitalopram	SSRI	0.3 (0.1)	0.7 (0.2)	0.7 (0.3)	0.3 (0.0)	Negative	Not involved	Not involved
Lorazepam	BZD	‐	‐	‐	‐	‐	+2	+2
Maprotiline	TeCA	0.1 (0.0)	0.2 (0.1)	3.0 (0.8)	0.3 (0.1)	Positive	Not involved	Not involved
Midazolam	BZD	‐	‐	‐	‐	‐	+2	+2
Sertraline	SSRI	3.9 (0.8)	1.4 (0.1)	0.8 (0.1)	0.2 (0.0)	Positive	Not involved	Not involved
Trazodone	SSRI	0.4 (0.2)	0.7 (0.1)	1.6 (0.6)	1.2 (0.2)	Negative	Not involved	+6
Venlafaxine	SNRI	0.8 (0.2)	2.0 (0.2)	4.0 (0.9)	2.7 0.1)	Positive	+10	Not involved
Vortioxetine	SSRI	0.5 (0.0)	0.3 (0.0)	0.4 (0.1)	0.3 (0.1)	Negative	Not involved	+10

Abbreviations: BZD, benzodiazepine; NDRI, norepinephrine‐dopamine reuptake inhibitor; RUCAM, Roussel Uclaf Causality Assessment Method; SD, Standard Deviation; SNRI, serotonin and norepinephrine reuptake inhibitor; SSRI, selective serotonin reuptake inhibitor; TCA, Tricyclic antidepressant; TeCA, Tetracyclic antidepressant.

^a^ Before hospitalization.

^b^ During hospitalization.

## 3 DISCUSSION AND CONCLUSIONS

Liver injury can be caused by different factors such as infections, toxic substances, autoimmunity, and drugs. DILI is considered when a pharmacological cause is suspected, and other causes have been discarded. Two pathological types of DILI have been identified. The first category is predictable and dose‐dependent. The second is idiosyncratic, slightly dose‐dependent, and unpredictable. It is the consequence either of immune‐related liver damage or of direct cellular injury.^7^ Antidepressant‐associated DILI is generally hepatocellular type and less frequency of the cholestatic or mixed types. The mechanism of DILI associated with antidepressants was thought to be metabolic or immuno‐allergic. In most cases, the onset of DILI is between several days and 6 months after the beginning of antidepressant treatment. A short latency of less than a month or clinical hallmarks (fever, rash, eosinophilia, autoantibodies) suggests an immunologic mechanism.^8^ Causality algorithms can help in the diagnosis of the culprit drug of liver damage, assigning a score to the suspected drugs.^9^ RUCAM‐based assessment has shown high sensitivity (86%), specificity (89%), positive predictive value (93%), and negative predictive value (78%),^10^ for a score between −1 to +4 for the non‐culprit drugs and +6 to +13 for the culprit drugs. RUCAM has been used to evaluate the causative drugs of DILI in inpatients at a single medical center in Korea. Antidepressants, antihistamines, and antibacterials were the common causative medicines for hepatotoxicity.^11^ However, RUCAM has poor discrimination when it is used in polypharmacy settings as that of our case (four drugs with a score ≥ +6). Very few drugs cause specific laboratory abnormalities. For instance, specific autoantibodies were reported against cytochrome P450 (CYP) such as CYP2C9 (by tienilic acid, not anymore marketed), CYP1A2 (by dihydralazin), CYP3A4 (by antiepileptic drugs), and CYP2E1 (by halothane).^12^ Therefore, alternative approaches are needed. LTT has been widely used in Japan for the diagnosis of DILI. Although some problems of LTT such as the presence of false positive and false negative cases have been reported,^13^ a new Japanese diagnostic scale adds +2 point for positive LTT cases to RUCAM.^14^ At first DILI episode, our results demonstrated a specific T‐cell reactivity to venlafaxine, supporting the algorithm and providing evidence of the type of mechanism involved. At 2^nd^ episode, LTT provided evidence of cellular immune response to one of three drugs involved by RUCAM. A case of DILI caused by ipragliflozin and assessed by RUCAM and LTT has been previously reported.^15^ Other authors have found that LTT seems to be a reliable test for diagnosing DILI.^16^, ^17^ However, disappointed results were reported using a modified LTT with readouts for soluble mediators (interleukins (IL)‐2, IL‐5, IL‐13, interferon −γ and granzyme B),^18^ Adopting LTT as a routine test in clinical laboratories is complicated because it is relatively technical demanding and difficult to reproduce.^6^ Furthermore, studies on standardization and validation of the test are necessary to determine its putative clinical utility.

Finally, LTT results with antidepressants not involved in DILI episodes have helped to select another clinically effective and safe treatment. Predictive values, specificity, and sensitivity of LTT in DILI induced by antidepressants are unknown. Therefore, we cannot interpret accurately the meaning of the pattern of LTT reactivity of our patient. There is not a straightforward relationship between drug exposure and LTT positivity since not all antidepressants to which the patient has been exposed give a positive LTT. On the other hand, our results also show that an antidepressant to which the patient has not previously been exposed, and with a negative LTT, has been an appropriate choice as an alternative medication (escitalopram). Chemical structure of antidepressants varies even within the same class (ie, SSRI), making it difficult to anticipate LTT results solely based on this factor. Clomipramine and amitriptyline, for instance, show different degrees of T‐cell proliferation despite sharing a tricyclic core. As for the side chain, primary amine may be involved in the T‐cell reactivity since clomipramine, maprotiline, and sertraline contain a primary amine in their structure and trigger cell proliferation. However, escitalopram has also a primary amine and does not induce a positive LTT. It would appear that immunogenicity of antidepressants in this patient might depend on the whole structure of the drug (rings and side chains), as it has been proposed for tricyclic antidepressant and aromatic anticonvulsants, or their metabolites.^19^ Therefore, our results suggest that LTT may be useful in polypharmacy settings and in selecting a future safety alternative drug for patients with DILI by antidepressants. The utility of LTT in polymedicated patients has also been previously proposed by others.^17^

To conclude, LTT combined with RUCAM algorithm may make treatments safer and more individualized in the event of an adverse drug reaction. Further research should be conducted to establish the performance and the cost‐effectiveness of this technique.

## CONFLICT OF INTEREST

The authors declare that they have no conflicts of interest.

## AUTHOR CONTRIBUTIONS

MGM: made a substantial contributions to conception and design, acquisition of data, and interpretation of data; involved in drafting the manuscript and revising it critically for important intellectual content. JMV: made a substantial contribution to the acquisition of data and analysis; involved in drafting the manuscript. EMS: made a substantial contributions to analysis, and interpretation of data; involved in and revising the manuscript critically for important intellectual content. SS: made a substantial contribution to the acquisition of data and analysis; and involved in drafting the manuscript. BBR: made a substantial contributions to analysis, and interpretation of data; involved in and revising the manuscript critically for important intellectual content. JM: made a substantial contributions to analysis, and interpretation of data; involved in and revising the manuscript critically for important intellectual content. LMS: made a substantial contribution to the acquisition of data and analysis; involved in drafting the manuscript. ESM: made a substantial contribution to the acquisition of data and analysis; involved in drafting the manuscript. ER: made a substantial contributions to conception and design, acquisition of data, and interpretation of data; involved in drafting the manuscript and revising it critically for important intellectual content.
